# Genomic disparity impacts variant classification of cancer susceptibility genes in Turkish breast cancer patients

**DOI:** 10.1002/cam4.6852

**Published:** 2024-02-02

**Authors:** Nihat B. Agaoglu, Busra Unal, Connor P. Hayes, McKenzie Walker, Ozden Hatirnaz Ng, Levent Doganay, Nisan D. Can, Huma Q. Rana, Arezou A. Ghazani

**Affiliations:** ^1^ Department of Medical Genetics, Division of Cancer Genetics Umraniye Training and Research Hospital Istanbul Turkey; ^2^ Division of Genetics Brigham and Women's Hospital Boston Massachusetts USA; ^3^ Department of Medical Biology, School of Medicine Acibadem University Istanbul Turkey; ^4^ Department of Molecular Biology Genetics and Biotechnology Istanbul Technical University Istanbul Turkey; ^5^ Division of Cancer Genetics and Prevention Dana‐Farber Cancer Institute Boston Massachusetts USA; ^6^ Department of Medicine Brigham and Women's Hospital Boston Massachusetts USA; ^7^ Harvard Medical School Boston Massachusetts USA

**Keywords:** breast cancer, cancer genetics, molecular genetics, next‐generation sequencing

## Abstract

**Objective:**

Turkish genome is underrepresented in large genomic databases. This study aims to evaluate the effect of allele frequency in the Turkish population in determining the clinical utility of germline findings in breast cancer, including invasive lobular carcinoma (ILC), mixed invasive ductal and lobular carcinoma (IDC‐L), and ductal carcinoma (DC).

**Methods:**

Two clinic‐based cohorts from the Umraniye Research and Training Hospital (URTH) were used in this study: a cohort consisting of 132 women with breast cancer and a non‐cancer cohort consisting of 492 participants. The evaluation of the germline landscape was performed by analysis of 27 cancer genes. The frequency and type of variants in the breast cancer cohort were compared to those in the non‐cancer cohort to investigate the effect of population genetics. The variant allele frequencies in Turkish Variome and gnomAD were statistically evaluated.

**Results:**

The genetic analysis identified 121 variants in the breast cancer cohort (actionable = 32, VUS = 89) and 223 variants in the non‐cancer cohort (actionable = 25, VUS = 188). The occurrence of 21 variants in both suggested a possible genetic population effect. Evaluation of allele frequency of 121 variants from the breast cancer cohort showed 22% had a significantly higher value in Turkish Variome compared to gnomAD (*p* < 0.0001, 95% CI) with a mean difference of 60 times (ranging from 1.37–354.4). After adjusting for variant allele frequency using the ancestry‐appropriate database, 6.7% (5/75) of VUS was reclassified to likely benign.

**Conclusion:**

To our knowledge, this is the first study of population genetic effects in breast cancer subtypes in Turkish women. Our findings underscore the need for a large genomic database representing Turkish population‐specific variants. It further highlights the significance of the ancestry‐appropriate population database for accurate variant assessment in clinical settings.

## 1 INTRODUCTION

Breast carcinoma is the leading cause of cancer mortality in women in Turkey, largely due to a lack of breast cancer awareness and delayed diagnosis.^1^ The most common histological type of cancer is ductal carcinoma (DC),^2^ followed by lobular carcinoma (LC).^3^ Women diagnosed with LC are more likely to have bilateral breast carcinoma and a first‐degree relative affected with breast carcinoma.^4^ Although there are well‐known breast cancer predisposition genes, most studies have been performed primarily on ductal breast carcinoma.^5^ The genetic susceptibility to lobular breast carcinoma is relatively unknown.^6^ *CDH1* is the only well‐known gene associated with increased invasive lobular carcinoma (ILC) risk.^7^, ^8^ Recently, *CHEK2* and *BRCA2* have been reported in association with ILC.^9^, ^10^

While studies have evaluated the genetic etiology of constitutional breast cancer in the Turkish population,^11^, ^12^ the effect of population genetics in the clinical evaluation of breast cancer in Turkish individuals remains unknown. The Turkish population is genetically unique due to a high rate of consanguineous marriage and admixture,^13^, ^14^, ^15^ which in turn can greatly affect the frequency of alleles in this population. The accurate assessment of allele frequency is critical in the clinical assessment of variant classification and determination of pathogenicity. Despite its value, the Turkish genome is underrepresented in publicly available databases. gnomAD, currently the largest genome database (v2 *n* = 125,748 exomes and 15,708 genomes, v3.1 *n* = 76,156 genomes), does not contain sub‐population data from Turkish individuals.^16^ In the Great Middle East (GME) Variome, only 12% (140/1111) of samples sequenced for the database are from the Turkish population.^17^ Turkish Variome is the only genome database of the Turkish population, it only contains 3362 combined exomes and genomes.^14^

Currently, Turkish Variome is the only publicly available database generated with the genome data obtained from Turkish individuals.^14^ It includes exome and genome data from non‐cancer patients with amyotrophic lateral sclerosis, ataxia, delayed sleep phase disorder, essential tremor, obesity, Parkinson's disease, polycystic ovarian syndrome, and neurological and immunological disorders.^14^

Turkish Variome is the only genome database of the Turkish population, it only contains 3362 combined exomes and genomes.

The effect of genomic inequity in population databases on patient's clinical management is well documented. Genomic inequity refers to an unequal opportunity for genetic testing or database generation in many geographical locations, due to economic and other limiting reasons, which in turn leads to the underrepresentation of many ethnic populations in large public genome databases. Recent studies highlighted the role of inadequate representation of population‐specific variants in current databases in the misclassification of variants in disease.^18^, ^19^ The underrepresentation of non‐European variants in databases has also been shown to be an incorrect result in a high percentage of variants of uncertain significance (VUS).^20^, ^21^ These limitations can perpetuate healthcare disparities further by resulting in inaccurate diagnoses.

This study aims to provide insight into the genetic background of invasive ductal carcinoma (IDC) and ILC patients from the Turkish population, focusing on germline findings and clinicopathological features. We hypothesized that some features of germline variants in our patient populations might be a function of genomic structure specific to the Turkish population. We addressed the effect of population‐specific allele frequency in the clinical assessment of germline sequence variants by performing a comparative evaluation of genetic variations in breast cancer and a non‐cancer control group from Turkey. The study highlights the importance of population‐specific databases to promote the unbiased application of genomic medicine.

## 2 PARTICIPANTS, MATERIALS AND METHODS

To test this hypothesis of the population effect on germline variations, we compared the germline findings of cancer and non‐cancer cohorts and compared the population allele frequency of the variants obtained from gnomAD with those obtained from Turkish Variome. The specific methods are described below.

### 2.1 Study cohort

#### 2.1.1 Breast cancer cohort

The breast cancer cohort included 43 patients with ILC, 69 with IDC, 3 with ductal carcinomas in situ (DCIS), and 17 with mixed invasive ductal and lobular carcinoma (IDC‐L). IDC‐L is poorly studied; as per the WHO classification, tumors with specialized histologic patterns occur in at least 50%–90% of the tumor area, and those with a non‐specialized pattern occur in 10%–49% of the tumor area.^22^ Data were collected from patients evaluated at Umraniye Research and Training Hospital (URTH) in 2020 and 2021. The age range of the breast cancer cohort was 24–80 years old. Before ordering the genetic testing, patients' medical and family histories were evaluated by clinical geneticists according to the current National Comprehensive Cancer Network (NCCN) guidelines.^23^ Patients with breast cancer who met the NCCN criteria were enrolled in the study. Patients without breast cancer and those who did not consent to genetic testing as part of the routine clinical practice were excluded from this study. Cascade testing for the families was offered and performed whenever it was possible, as part of routine clinical management. Informed consent was obtained from the patients as part of the routine clinical care.

#### 2.1.2 Non‐cancer cohort

The non‐cancer control cohort comprised a total of 492 participants. The participants were recruited as part of a different study in the URTH between 2016 and 2017. The inclusion criteria were the absence of personal and family history of cancer as assessed by the clinical geneticist and research associate before genetic testing. None of these individuals had a personal or family history of cancer, but some presented with hypertension and diabetes mellitus. The probands in the non‐cancer control were unrelated. The age range of individuals in the non‐cancer cohort was between 62 and 104 years old. Participants without a genetic panel test results were excluded from this study. Cascade testing and routine clinical management were offered to the non‐cancer cohort with any unexpected pathogenic (P)/likely pathogenic (LP) findings. Participants consented to a broad research study per institutional ethical policy at URTH.

### 2.2 Sample preparation, next‐generation sequencing, and bioinformatics

The genomic DNA was extracted from peripheral blood by QIAamp DNA Mini QIA cube Kit (Qiagen, Hilden, Germany) before library preparation as previously described.^11^, ^24^ The extracted DNA was run on a 27 gene panel which was the current gene panel at URTH during the study period (approved by the Ministry of Health in Turkey). Briefly, Sophia Hereditary Cancer Solution kit (Sophia Genetics, Lausanne, Switzerland) consisting of coding regions of 27 genes (*ATM*, *BARD1*, *BRCA1*, *BRCA2*, *BRIP1*, *CDH1*, *CHEK2*, *FAM175A*, *MRE11A*, *NBN*, *PALB2*, *PIK3CA*, *RAD50*, *RAD51C*, *RAD51D*, *TP53*, *XRCC2*, *MLH1*, *MSH2*, *MSH6*, *EPCAM*, *PMS2*, *PMS2CL*, *MUTYH*, *APC*, *PTEN*, and *STK11*) was used for library preparation. Sequencing was performed by NextSeq500 platform (Illumina Inc., San Diego, USA). FASTQ data were analyzed by SOPHiA Data‐Driven Medicine (Sophia DDM, Sophia Genetics v4.2, hg19 alignment). Copy numbers were identified by measuring the coverage levels of the desired regions along with samples within the same run.^25^

### 2.3 Development of genetic database of Turkish participants

A genetic variant database for all 625 participants (non‐cancer *n* = 492, breast cancer *n* = 132) was generated. For the cancer cohort, personal cancer history, demographic information, three‐generation family cancer history, and histopathological features of the carcinoma types were included. For all participants, details of germline variants and supported evidence were included in the database. ACMG classification^26^ was performed for all variants, and designations of P, LP, and VUS were included. gnomAD (v2.1.1 *n* = 125,748 exomes and 15,708 genomes) database was used to investigate allele frequency in large population databases.^16^ Turkish Variome (*n* = 2589 exomes and *n* = 773 genomes) was used to investigate allele frequency, more specifically in the Turkish population.^14^

### 2.4 Query of database

The genes identified in the cancer cohort were compared with the non‐cancer cohort to evaluate the population genetics effect. In addition, the occurrence of each variant from the cancer cohort was crosschecked with the non‐cancer cohort. The denoted variant allele frequencies of the VUS in Turkish Variome and gnomAD were analyzed. These variants were reevaluated for variant classification with consideration of their allele frequencies in these databases.

### 2.5 Statistical analysis

The allele frequency of all 121 variants from the breast cancer groups was obtained from Turkish Variome and gnomAD. The differences in allele frequencies in the 2 groups were measured using Prism, unpaired *t*‐test (GraphPad, version 9.5). The two‐tailed *p* value was calculated with a 95% confidence interval.

## 3 RESULTS

### 3.1 Patient demographics and clinicopathologic characteristics

A total of 60 patients were histopathologically diagnosed with ILC (*n* = 43) and IDC‐L (*n* = 17). All patients were female, with a median age of 44 (range 24–80 years) (Table 1). In four patients, multiple primary carcinomas were detected in addition to lobular breast carcinoma. Nine patients presented with bilateral breast carcinoma. ER and PR positivity rate was 83.3% and 81.6%, respectively, whereas 16.6% of the ILC and IDC‐L cases were HER2 positive (Table 1). Of 60 patients, 36 had available E‐cadherin test results, of which 34 were negative. Family history assessment showed 39 patients (65%) had positive family history of cancer, including breast cancer, in the first‐degree relatives. In 38 patients (63.3%) the family history extended to the second‐degree relatives, and in 11 patients (18.3%) to the third‐degree relatives (Tables S1 and S2).

**TABLE 1:** Demographic and tumor characteristics of patients with breast carcinoma.

Patient and tumor characteristics	Invasive lobular carcinoma/mixed invasive ductal and lobular carcinoma of the breast	Ductal breast carcinoma
Number of patients	ILC:43	72
	IDC‐L:17	
Male	0 (0%)	0 (0%)
Female	60 (100%)	72 (100%)
Median age at diagnosis	44 (24–80)	45 (26–70)
<40 age number of patients	13 (21.7%)	20 (28%)
≥40 age number of patients	48 (78.3%)	52(72%)
Histopathologic type		
Invasive ductal carcinoma		69 (95.8%)
Ductal carcinoma in situ		3 (4.2%)
Invasive lobular carcinoma	43 (71.7%)	
Mixed invasive ductal and lobular carcinoma	17 (28.3%)	
Multiple primary cancer	4 (6.6%)	2 (2.8%)
Receptor positivity		
ER	50 (83.3%)	53 (73.6%)
PR	49 (81.6%)	51 (70.8%)
HER‐2	10 (16.6%)	23 (32%)
Unknown	5 (8.3%)	6 (8.1%)
E‐cadherin expression		
Positive	2 (3.3%)	N/A
Negative/low expression	34 (56.7%)	N/A
Unknown	24 (40%)	N/A

Abbreviations: ER, estrogen receptor; HER2, human epidermal growth factor receptor; IDC‐L, mixed invasive ductal and lobular carcinoma of the breast; ILC, invasive lobular carcinoma; PR, progesterone receptor.

Patients with ductal breast carcinoma (IDC *n* = 69, DCIS *n* = 3) had a median age of 45 (26–70 years); 20 of these patients were younger than 40 years old (Table 1). The ER, PR, and HER‐2 positivity rates were 73.6%, 70.8%, and 32%, respectively (Table 1). The evaluation of family history showed that 57 (79.2%) patients had a positive family history of cancer, including breast cancer, in the first‐degree relatives. In 64 (88.8%) patients, the positive family history extended to the second‐degree family members, and in 28 patients (38.9%) to the third‐degree relatives (Table S3).

The non‐cancer group included 492 participants, 308 of whom were female and 184 were male. The average age of participants was 75, ranging from 65 to 104 years old. All participants were negative for any personal and family history of cancer (Table S4).

### 3.2 Genomic analysis of breast cancer cohort

**FIGURE 1:**
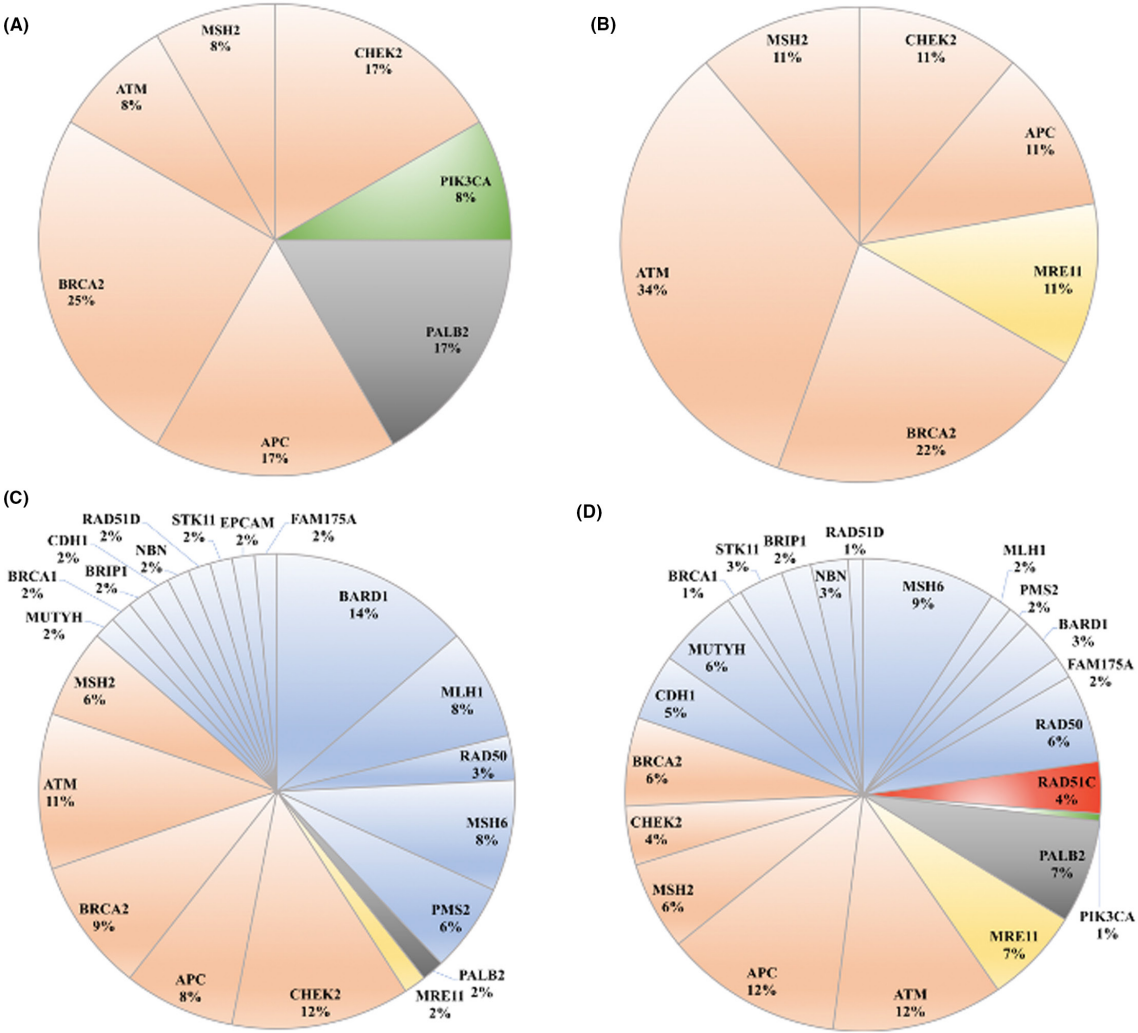
(A) Distribution of cancer genes harboring reported variants in patients diagnosed with ILC, (B) IDC‐L, (C) IDC, and (D) in the non‐cancer cohort. Orange color represents commonly affected genes in all these four groups. Gray represents shared genes between (A), (C), and (D). The yellow color represents shared genes between (B), (C), and (D). Green and blue colors represent common genes between (A) and (D) and between (C) and (D), respectively. Red represents a unique gene in the non‐cancer control cohort.

The genetic assessment of 43 females with ILC showed that four patients (9%) had actionable variants in *CDH1*, *BRCA2*, *MUTYH*, *RAD50*, and *CHEK2* (Table S1). There were no recurring variants among these patients, except for *CHEK2* c.1427C > T, a known common risk factor. There were 13 VUS reported in this group, more commonly in *BRCA2*, followed by *APC*, *CHEK2*, and *PALB2* (Figure 1). In our IDC‐L cohort, two patients harbored P, LP variants in *MSH2*, and *PALB2*. Nine VUS were identified in the IDC‐L cohort (Table S2). The most common gene harboring VUS was *ATM*, followed by *BRCA2* (Figure 1).

The genetic evaluation of the 72 patients with IDC revealed that 20 (27.8%) patients were positive for P, LP variants in *BRCA2*, *NBN*, *ATM*, *BRCA1*, *CHEK2*, *MLH1*, *MSH6*, *PALB2*, and *TP53* genes (Table S3). *BRCA1* (*n* = 5) and *BRCA2* (*n* = 5) were the most frequently mutated genes, as commonly reported in ductal breast carcinoma. Overall, 56 patients harbored VUS more frequently in *BARD1* and *CHEK2* (Figure 1). A total of 34 (61%) VUS were in the genes without a strong association with breast cancer.

Actionable results among the ILC, IDC‐L, and IDC cohorts were observed in *BRCA2*, *PALB2*, *MUTYH*, *RAD50*, *CDH1*, and *MSH2* genes. The more diverse genes were observed in the IDC cohort (Table S3). Notably, *BRCA1* was only affected in the IDC group. At the variant level, there were no common VUS among these breast cancer groups (Table S5), except for *CHEK2* c.1427C > T (classified as a risk factor) and *CHEK2* c.549G > C (VUS). In total, 75 non‐recurrent VUS were found in the breast cancer cohort (Table S5). The distribution of genes with VUS designation was similar in the three breast cancer groups (Figure 1). The occurrence of VUS in these three breast cancer subtypes might be a factor in population genetic structure. The distribution and frequency of the VUS from the breast cancer group were therefore evaluated against those from the non‐cancer cohort collected from the same center, presumably all representing the Turkish population at large.

### 3.3 Genome analysis of non‐cancer cohort

In the non‐cancer cohort, 25/492 (5%) participants carried 17 unique P/LP variants in *ATM*, *BRCA2*, *BRIP1*, *CHEK2*, *MUTYH*, *NBN*, and *PMS2* genes (Table S4). *MUTYH* (*n* = 9) followed by *CHEK2* (*n* = 6) were the most affected genes. None of the P, LP variants in the control cohort was seen in the breast cancer cohort. Regarding VUS distribution, 166/492 (34%) participants had VUS in 21 genes (Figure 1), with *APC* and *ATM* being the most involved genes (*n* = 24).

### 3.4 The analysis of population genetics effects

We hypothesized that the occurrence of the germline variants in our patients might be a function of genomic structure specific to the Turkish population. To test this hypothesis, we compared the germline findings identified in the cancer and non‐cancer cohorts. At the variant level, 21 variants were common between the breast cancer and non‐cancer cohorts (Table S5).

**FIGURE 2:**
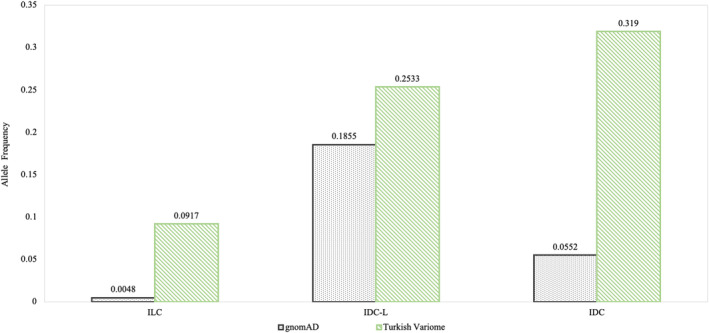
The range of population allele frequencies of VUS in cancer genes identified in three breast cancer subgroups. The ratio of variant allele frequency in Turkish Variome over gnomAD was 19 in invasive lobular carcinoma (ILC), 1.36 in mixed invasive ductal and lobular carcinoma (IDC‐L), and 6 in invasive ductal carcinoma (IDC). The green bar represents variant allele frequency in the Turkish Variome. The dotted bar represents variant allele frequency in gnomAD.

**FIGURE 3:**
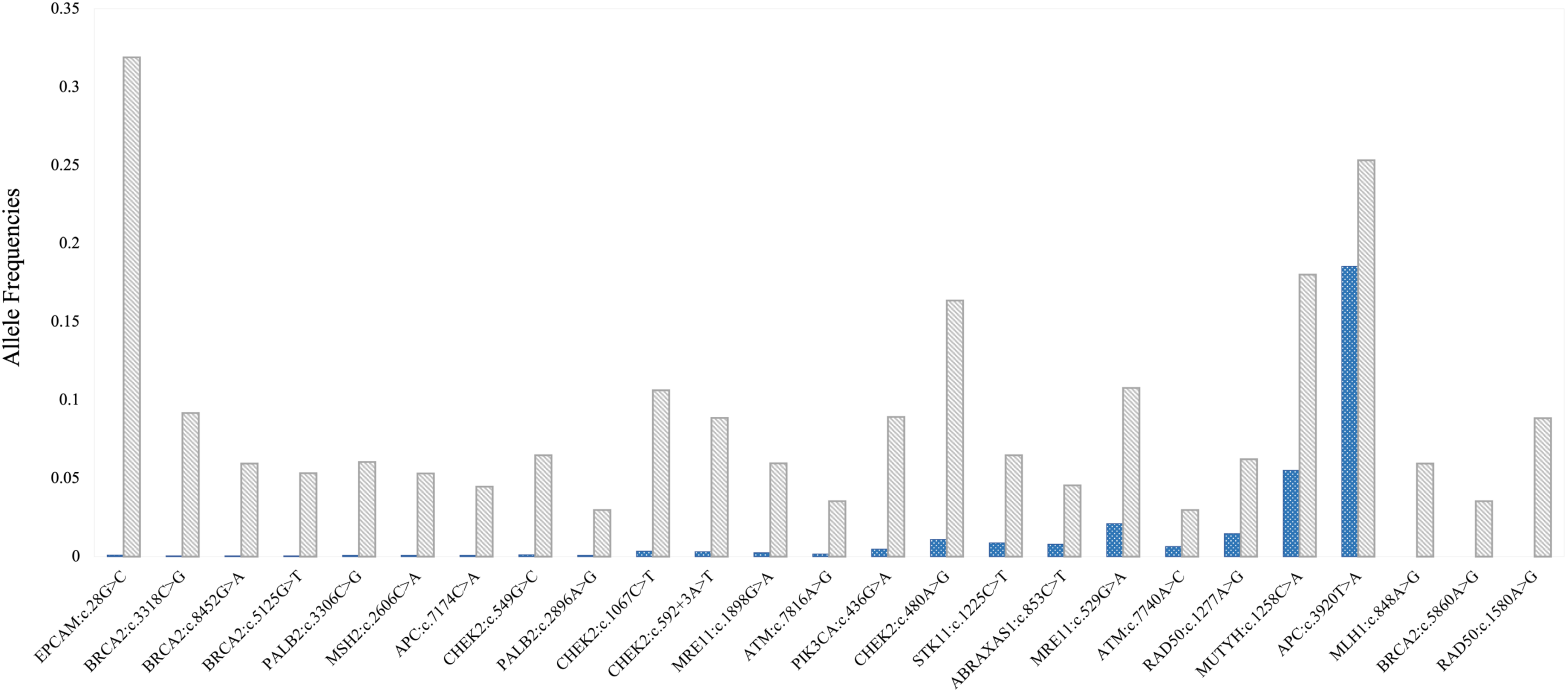
List of variants of uncertain significance VUS in three breast cancer cohorts and associated allele frequencies. The allele frequency obtained from Turkish Variome is shown in gray, and from gnomAD is shown in blue.

**FIGURE 4:**
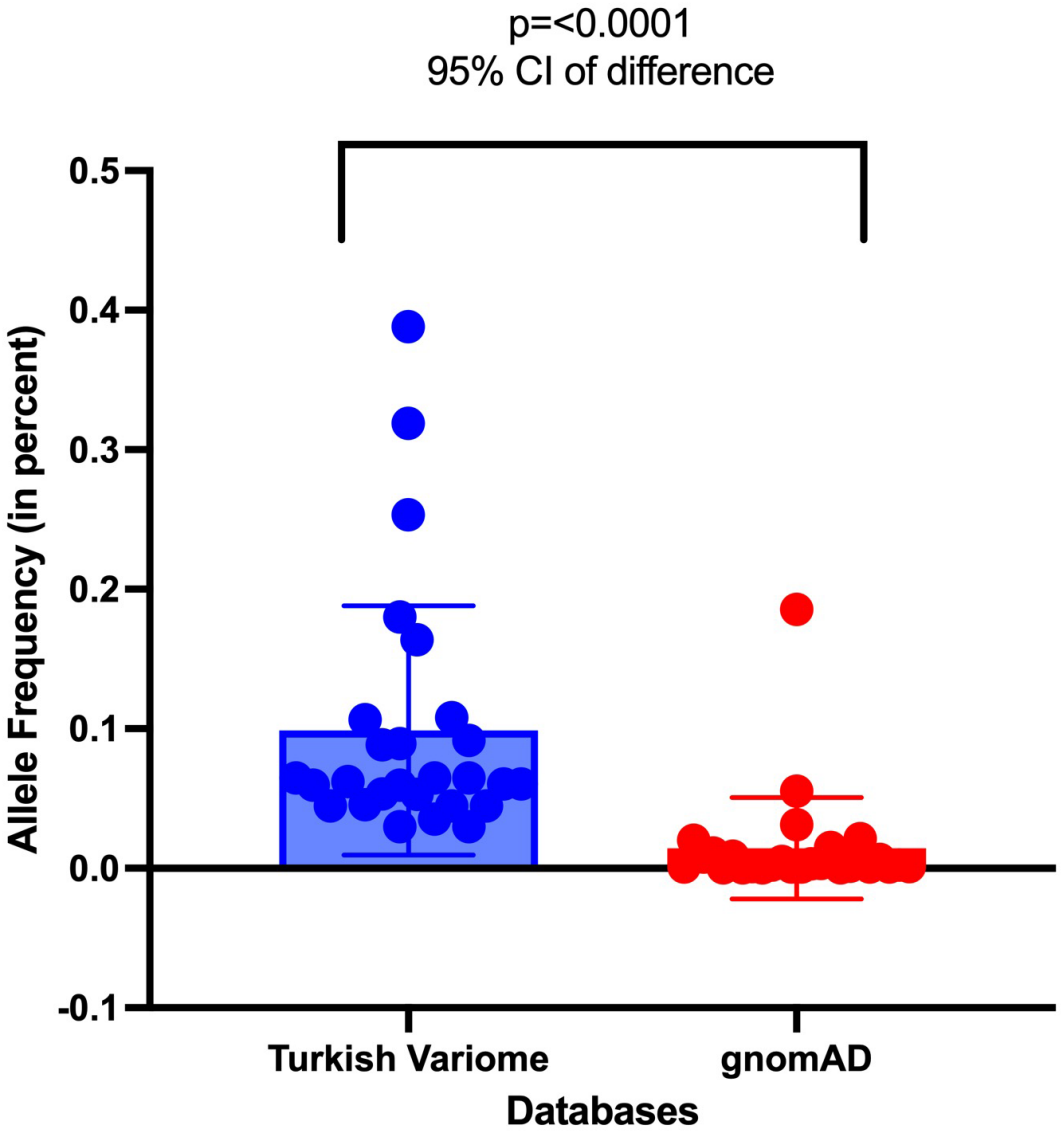
Comparative analysis of variant allele frequency values in gnomAD and Turkish Variome. The differences in allele frequencies of 121 variants in the 2 groups were measured using an unpaired *t*‐test in Prism (GraphPad, version 9.5). The two‐tailed *p* value was calculated with a 95% confidence interval.

To assess the effect of population genetic structure on a larger scale, the allele frequency of VUS was assessed using both gnomAD and Turkish Variome. The gnomAD is the largest population genome database, but it does not contain Turkish subpopulation data. Turkish Variome, while much smaller, is a genome database generated from the Turkish genomic non‐cancer data. In the ILC patient group, the aggregated allele frequency of VUS in the Turkish Variome was 19 times larger than the value in gnomAD; in IDC‐L and IDC patients this allele frequency difference was respectively 1.36 times and 6 times greater (Figure 2). Altogether of 75 VUS in breast cancer groups, 25 variants (33%) had higher allele frequency in the Turkish Variome than in gnomAD (Figure 3). A total of 22 VUS had an allele frequency reported both in gnomAD and Turkish Variome; the allele frequency in 100% of these variants was greater in Turkish Variome compared to those in gnomAD (on average 60 times, ranging from 1.37–354.4 times) (Table S5). A total of 5/25 P, LP variants were reported both in gnomAD and Turkish Variome, all had greater frequency in Turkish Variome compared to gnomAD, on average 49 times, ranging from 2.2 to 161.74. Overall, the allele frequency value of 27 variants (VUS *n* = 22, P, LP *n* = 5) was significantly higher in Turkish Variome compared to those in gnomAD (*p* < 0.0001, 95% CI, Figure 4).

We further set to investigate the effect of population allele database in variant classification. We conducted ACMG classifications by using both the allele frequencies in Turkish Variome and gnomAD. PM2, the evidence supporting the absence or extremely rare allele frequency, was invoked for 74 variants based on values in gnomAD (Table S5). Using allele frequency in the Turkish Variome, however, only 59 variants met the PM2 criterion and 2 met the BS1 criterion, due to a more common occurrence of variants in the Turkish Variome. Overall, this affected the classification of 6.7% (5/75) of variants, as they were downgraded from VUS to likely benign (Table S5). The variants were in *EPCAM*, *RAD50*, *STK11*, and *MRE11* genes. The allelic frequencies of the actionable variants were below the PM2 threshold in both Turkish Variome and gnomAD (Table S6).

## 4 DISCUSSION

We addressed two goals in this study. First, we aimed to assess the population‐specific germline etiology of lobular and ductal breast carcinoma in the Turkish population. To that end, we performed a comprehensive investigation of patients with lobular and ductal breast carcinomas from the Turkish population by assessing germline genetic, clinical, and histopathological features. Second, we investigated the effect of population genetics in variant classification by assessing germline variants detected in 132 patients with all subtypes of breast cancer and those detected in 492 individuals in the non‐cancer group.

Histopathological features of our breast cancer cohort from the Turkish population were similar to those reported previously in non‐Turkish individuals. ILC and IDC pathology findings are generally ER+, PR+, and HER‐2‐status.^27^ Although the current studies on IDC‐L characteristics are scarce and consist of small cohort sizes, the immunohistochemical profile of IDC‐L is found to be similar to ILC.^28^ The lack of E‐cadherin expression is the hallmark of ILC and IDC‐L.^29^ In concordance with these studies, the majority of our patients with ILC, IDC‐L, or IDC were ER‐positive, PR‐positive, and HER‐2‐negative. As expected, E‐cadherin was negative in 34 out of 36 patients with available E‐cadherin test results in our ILC and IDC‐L cohorts. Almost all studies showed ER and PR positivity is more likely in ILC and IDC‐L than IDC.^27^ This was in line with the higher ER and PR positive rates among ILC/IDC‐L patients than IDC/ DCIS in our cohort. The bilaterality of breast cancer was 15% and 4% in ILC/IDC‐L and DCIS/IDC cohorts, respectively, and these results are consistent with previous studies defining lobular breast carcinoma as a possible risk factor for the onset of bilateral breast cancer.^30^

Around 10%–15% of breast cancers are familial, and this rate is shown to be higher in ILCs.^4^ In our Turkish breast cancer cohort, 27% of our patients with ILC or IDC‐L had a first‐degree relative affected by breast cancer. Germline variants of *CDH1* are described in ILC.^31^, ^32^ However, we only detected one patient with germline pathogenic *CDH1* variant, which might stem from the small patient number in our ILC cohort. Previous studies have reported *CDH1* and *BRCA2* (and not *BRCA1* and *CHEK2* c.470 T > C) to be strongly associated with ILC.^10^, ^33^ Despite the small sample size, we observed similar findings; the *BRCA2* pathogenic variant, but not the *BRCA1* and *CHEK2* c.470 T > C, were present in our ILC cohort. The *BRCA1*, *BRCA2*, *TP53*, *CHEK2*, *ATM*, and *PALB2* genes are well‐known for predisposition to IDC.^5^ In our IDC/DCIS cohort, in addition to these genes, we identified P, and LP variants in *MSH6* and *MLH1*. The risk association between these genes and breast cancer is not well established.^34^

The population allele frequency is a main criterion for assessing the pathogenicity of variants per the ACMG guideline. In the currently available genome databases, 59%–94% of sequence data are obtained from European individuals.^16^, ^35^, ^36^ In one study of the Brazilian population, 207,621 variants identified in their cohort were not reported in major publicly available genomic databases.^37^ Therefore, these databases may not adequately represent the genomic diversity of underrepresented populations, such as the Turkish population. The genomic diversity of the Turkish population is well‐documented. Every subregion of Turkey has a diverse admixture, mainly consisting of four groups of population of Europe, Balkan, Caucasus, and GME.^14^ Inbreeding also plays a crucial role in the genetics of the Middle Eastern populations.^13^ Turkey's average consanguineous marriage rate is 21.1%.^38^ This high rate of consanguinity may increase the occurrence of excessively rare variants,^14^ as well as the occurrence of common pathogenic or polymorphic variants in Turkish individuals compared to other populations. In our non‐cancer control cohort, 6% of the variants were present in the breast cancer cohort. This observation might be due to the population‐specific structure. However, the common occurrence of these variants could be explained by reduced or incomplete penetrance of alleles. Further large‐scale studies are required to delineate the underlying effect of population genetics in cancer cohorts.

Currently, Turkish Variome is the only publicly available database generated with the genome data obtained from Turkish individuals.^14^ It includes exome and genome data from non‐cancer patients with amyotrophic lateral sclerosis, ataxia, delayed sleep phase disorder, essential tremor, obesity, Parkinson's disease, polycystic ovarian syndrome, and neurological and immunological disorders.^14^ Despite the smaller size of the Turkish Variome compared to gnomAD, in our cancer cohort, 27/121variants had a significantly higher allele frequency in the Turkish Variome compared to gnomAD (*p* < 0.0001, 95% CI) the difference in ratio ranging from 1.37 to 354 times. The allele frequency in the Turkish population could potentially be higher when assessed in a larger genome database generated with sequencing data from healthy Turkish individuals. This highlights the critical role of population genetic structure in genetic medicine. It also emphasizes the need for a comprehensive genomic variant database specific to the Turkish population for the clinical evaluation of patients with Turkish backgrounds.

The accurate assessment of the clinical utility of variants is crucial in patient management. In our cancer cohort, 6.7% (5/75) of VUS were reclassified to likely benign by using Turkish Variome. Although downgrading VUS to LB might not often affect patient management, this reclassification holds clinical importance.^39^ There are reports of diverse practices of surgical procedures for patients with VUS in clinically relevant genes.^40^, ^41^, ^42^ Patients with reported VUS experience increased anxiety and even unnecessary procedures.^43^, ^44^ The clinical, economic, and psychosocial burden of VUS is well‐studied.^43^, ^45^, ^46^, ^47^ As genome sequence databases grow in volume, VUS can be more accurately assessed and perhaps reclassified. In a large study on the prevalence of variant reclassification, 91.2% of VUS assessed between 2006 and 2018 were downgraded to likely benign or benign.^48^ The reclassifications are feasible due to the accumulation of larger genome databases and additional supporting evidence. Similarly, variant reclassification has been reported in ancestry‐based studies in underrepresented and diverse ethnic backgrounds.^39^, ^49^, ^50^, ^51^, ^52^, ^53^ Therefore, large prospective genetic studies are needed to assess the larger reclassification of variants in Turkish and other populations. Furthermore, the development of large gnomAD‐like databases representing the Turkish population and making such knowledge bases publicly available is a crucial next step in addressing the current limitations. Future directions should include the assembly of aggregate allele data from healthy individuals as well as different disease groups, such as cancer, neurology, etc. This disease‐ and population‐specific collation of databases would allow the utility of an accurate allele frequency value in variant classifications and, therefore more informed clinical management of patients with specific genetic conditions. Data sharing is equally crucial in addressing genomic disparity. Making population‐specific genome aggregate databases publicly and freely accessible will allow clinicians and researchers to utilize the variant data.

While this study underscores the importance of ancestry‐appropriate databases in genomic medicine, several limitations should be acknowledged. Individuals without cancer diagnosis but with a strong family cancer history could not be included in the study. The patient cohort in the cancer genetics center at UTRH largely represents those who are referred to this center for cancer evaluation. Therefore, most patients have a personal history of cancer, with or without a family history. Another limitation may stem from the timeline when the personal and family cancer history of the non‐cancer cohort was collected. Given that data were collected in 2016–2017, some of this information might have changed over time. Additionally, the time difference in testing for the non‐cancer and cancer cohort was about 4–5 years. Although the number of genes in the testing panel remained the same, the informatics and annotation tools were updated to newer versions over time. To account for possible changes in annotations, all germline variants in this study were assessed for population allele frequency at the time of this study.

In conclusion, we comprehensively investigated the genetic susceptibility of breast cancer subtypes. To our knowledge, no other studies investigated these subtypes of breast cancer in the Turkish population. This study highlights the role of a population‐specific genome database in the genetic assessment of breast cancer in the Turkish population. The statistically significant difference of variant frequencies between gnomAD and Turkish Variome indicates the importance of creating and using ancestry‐appropriate genomic databases to decrease inequity in genomic medicine. Further, it shows the potential effect of genomic databases on clinical management by decreasing the reported VUS ratio. More globally, it presents the need for creating larger genomic databases from genetically underrepresented populations for patient care in addressing healthcare disparities in genomic medicine.

## AUTHOR CONTRIBUTIONS

**Nihat B. Agaoglu:** Data curation (lead); formal analysis (lead); investigation (lead); methodology (equal); writing – review and editing (equal). **Busra Unal:** Data curation (lead); formal analysis (lead); investigation (supporting); methodology (lead); writing – original draft (lead). **Connor Hayes:** Data curation (equal); formal analysis (equal); methodology (equal); writing – review and editing (equal). **McKenzie Walker:** Formal analysis (equal); methodology (equal); writing – review and editing (equal). **Ozden Hatirnaz Ng:** Investigation (equal); methodology (equal); writing – review and editing (equal). **Levent Doganay:** Data curation (equal); methodology (equal); writing – review and editing (equal). **Nisan D. Can:** Methodology (equal); writing – review and editing (equal). **Huma Q. Rana:** Investigation (equal); writing – review and editing (equal). **Arezou A. Ghazani:** Conceptualization (lead); data curation (equal); formal analysis (equal); investigation (equal); methodology (equal); project administration (lead); resources (lead); supervision (lead); validation (equal); writing – original draft (equal); writing – review and editing (lead).

## CONFLICT OF INTEREST STATEMENT

The authors declare no conflict of interest.

## ETHICS STATEMENT

The ethical committee of Umraniye Training and Research Hospital (No: 49/224.03.2016) and Acıbadem University and Acıbadem Healthcare Institutions Medical Research Ethics Committee (ATADEK‐2022/15 30.09.2022) approved this study and informed consent was obtained from all the participants.

## Supporting information


Table S1.

Table S2.

Table S3.

Table S4.

Table S5.

Table S6.

## Data Availability

Data available on request from the authors ‐ The data that support the findings of this study are available from the corresponding author upon reasonable request.

## References

[1] Özmen V , Özmen T , Doğru V . Breast cancer in Turkey; an analysis of 20.000 patients with breast cancer. Eur J Breast Health. 2019;15(3):141‐146. 31312788 10.5152/ejbh.2019.4890 PMC6619786

[2] Sung H , Ferlay J , Siegel RL , et al. Global cancer statistics 2020: GLOBOCAN estimates of incidence and mortality worldwide for 36 cancers in 185 countries. CA Cancer J Clin. 2021;71(3):209‐249. 33538338 10.3322/caac.21660

[3] Li CI , Anderson BO , Daling JR , Moe RE . Trends in incidence rates of invasive lobular and ductal breast carcinoma. JAMA. 2003;289(11):1421‐1424. 12636465 10.1001/jama.289.11.1421

[4] Claus EB , Risch N , Thompson WD , Carter D . Relationship between breast histopathology and family history of breast cancer. Cancer. 1993;71(1):147‐153. 8380113 10.1002/1097-0142(19930101)71:1<147::aid-cncr2820710124>3.0.co;2-v

[5] Mavaddat N , Dorling L , Carvalho S , et al. Pathology of tumors associated with pathogenic germline variants in 9 breast cancer susceptibility genes. JAMA Oncol. 2022;8(3):e216744. 35084436 10.1001/jamaoncol.2021.6744 PMC8796069

[6] Yadav S , Couch FJ . Germline genetic testing for breast cancer risk: the past, present, and future. Am Soc Clin Oncol Educ Book. 2019;39:61‐74. 31099663 10.1200/EDBK_238987

[7] Pharoah PD , Guilford P , Caldas C , International Gastric Cancer Linkage Consortium . Incidence of gastric cancer and breast cancer in CDH1 (E‐cadherin) mutation carriers from hereditary diffuse gastric cancer families. Gastroenterology. 2001;121(6):1348‐1353. 11729114 10.1053/gast.2001.29611

[8] Hansford S , Kaurah P , Li‐Chang H , et al. Hereditary diffuse gastric cancer syndrome: CDH1 mutations and beyond. JAMA Oncol. 2015;1(1):23‐32. 26182300 10.1001/jamaoncol.2014.168

[9] van Veen EM , Evans DG , Harkness EF , et al. Extended gene panel testing in lobular breast cancer. Fam Cancer. 2022;21(2):129‐136. 33763779 10.1007/s10689-021-00241-5 PMC8964550

[10] Yadav S , Hu C , Nathanson KL , et al. Germline pathogenic variants in cancer predisposition genes among women with invasive lobular carcinoma of the breast. J Clin Oncol. 2021;39(35):3918‐3926. 34672684 10.1200/JCO.21.00640 PMC8660003

[11] Akcay IM , Celik E , Agaoglu NB , et al. Germline pathogenic variant spectrum in 25 cancer susceptibility genes in Turkish breast and colorectal cancer patients and elderly controls. Int J Cancer. 2021;148(2):285‐295. 32658311 10.1002/ijc.33199

[12] Bora E , Caglayan AO , Koc A , et al. Evaluation of hereditary/familial breast cancer patients with multigene targeted next generation sequencing panel and MLPA analysis in Turkey. Cancer Genet. 2022;262–263:118‐133. 10.1016/j.cancergen.2022.02.006 35220195

[13] Yang X , Al‐Bustan S , Feng Q , et al. The influence of admixture and consanguinity on population genetic diversity in Middle East. J Hum Genet. 2014;59(11):615‐622. 25253659 10.1038/jhg.2014.81

[14] Kars ME , Başak AN , Onat OE , et al. The genetic structure of the Turkish population reveals high levels of variation and admixture. Proc Natl Acad Sci U S A. 2021;118(36):1‐10. 10.1073/pnas.2026076118 PMC8433500 34426522

[15] Akbayram S , Sari N , Akgün C , et al. The frequency of consanguineous marriage in eastern Turkey. Genet Couns. 2009;20(3):207‐214. 19852426

[16] Karczewski KJ , Francioli LC , Tiao G , et al. The mutational constraint spectrum quantified from variation in 141,456 humans. Nature. 2020;581(7809):434‐443. 32461654 10.1038/s41586-020-2308-7 PMC7334197

[17] Scott EM , Halees A , Itan Y , et al. Characterization of greater Middle Eastern genetic variation for enhanced disease gene discovery. Nat Genet. 2016;48(9):1071‐1076. 27428751 10.1038/ng.3592 PMC5019950

[18] Spratt DE , Chan T , Waldron L , et al. Racial/ethnic disparities in genomic sequencing. JAMA Oncol. 2016;2(8):1070‐1074. 27366979 10.1001/jamaoncol.2016.1854 PMC5123755

[19] Manrai AK , Funke BH , Rehm HL , et al. Genetic misdiagnoses and the potential for health disparities. N Engl J Med. 2016;375(7):655‐665. 27532831 10.1056/NEJMsa1507092 PMC5292722

[20] Caswell‐Jin JL , Gupta T , Hall E , et al. Racial/ethnic differences in multiple‐gene sequencing results for hereditary cancer risk. Genet Med. 2018;20(2):234‐239. 28749474 10.1038/gim.2017.96

[21] Roberts ME , Susswein LR , Janice Cheng W , et al. Ancestry‐specific hereditary cancer panel yields: moving toward more personalized risk assessment. J Genet Couns. 2020;29(4):598‐606. 32227564 10.1002/jgc4.1257

[22] Sinn HP , Kreipe H . A brief overview of the WHO classification of breast tumors, 4th edition, focusing on issues and updates from the 3rd edition. Breast Care (Basel). 2013;8(2):149‐154. 24415964 10.1159/000350774 PMC3683948

[23] Daly MB , Pal T , Berry MP , et al. Genetic/familial high‐risk assessment: breast, ovarian, and pancreatic, version 2.2021, NCCN clinical practice guidelines in oncology. J Natl Compr Canc Netw. 2021;19(1):77‐102. 33406487 10.6004/jnccn.2021.0001

[24] Agaoglu NB , Ng OH , Unal B , et al. Concurrent pathogenic variants of BRCA1, MUTYH and CHEK2 in a hereditary cancer family. Cancer Genet. 2022;268‐269:128‐136. 10.1016/j.cancergen.2022.10.144 36368126

[25] Agaoglu NB , Unal B , Akgun Dogan O , et al. Determining the accuracy of next generation sequencing based copy number variation analysis in hereditary breast and ovarian cancer. Expert Rev Mol Diagn. 2022;22(2):239‐246. 35240897 10.1080/14737159.2022.2048373

[26] Richards S , Aziz N , Bale S , et al. Standards and guidelines for the interpretation of sequence variants: a joint consensus recommendation of the American College of Medical Genetics and Genomics and the Association for Molecular Pathology. Genet Med. 2015;17(5):405‐424. 25741868 10.1038/gim.2015.30 PMC4544753

[27] Zhao H . The prognosis of invasive ductal carcinoma, lobular carcinoma and mixed ductal and lobular carcinoma according to molecular subtypes of the breast. Breast Cancer. 2021;28(1):187‐195. 32812198 10.1007/s12282-020-01146-4

[28] Bharat A , Gao F , Margenthaler JA . Tumor characteristics and patient outcomes are similar between invasive lobular and mixed invasive ductal/lobular breast cancers but differ from pure invasive ductal breast cancers. Am J Surg. 2009;198(4):516‐519. 19800459 10.1016/j.amjsurg.2009.06.005

[29] McCart Reed AE , Kutasovic JR , Lakhani SR , Simpson PT . Invasive lobular carcinoma of the breast: morphology, biomarkers and ‘omics’. Breast Cancer Res. 2015;17(1):12. 25849106 10.1186/s13058-015-0519-x PMC4310190

[30] Wang K , Zhu GQ , Shi Y , Li ZY , Zhang X , Li HY . Long‐term survival differences between T1‐2 invasive lobular breast cancer and corresponding ductal carcinoma after breast‐conserving surgery: a propensity‐scored matched longitudinal cohort study. Clin Breast Cancer. 2019;19(1):e101‐e115. 30502219 10.1016/j.clbc.2018.10.010

[31] Shenoy S . CDH1 (E‐cadherin) mutation and gastric cancer: genetics, molecular mechanisms and guidelines for management. Cancer Manag Res. 2019;11:10477‐10486. 31853199 10.2147/CMAR.S208818 PMC6916690

[32] Gall TM , Frampton AE . Gene of the month: E‐cadherin (CDH1). J Clin Pathol. 2013;66(11):928‐932. 23940132 10.1136/jclinpath-2013-201768

[33] Petridis C , Arora I , Shah V , et al. Frequency of pathogenic germline variants in CDH1, BRCA2, CHEK2, PALB2, BRCA1, and TP53 in sporadic lobular breast cancer. Cancer Epidemiol Biomarkers Prev. 2019;28(7):1162‐1168. 31263054 10.1158/1055-9965.EPI-18-1102

[34] Nikitin AG , Chudakova DA , Enikeev RF , et al. Lynch syndrome germline mutations in breast cancer: next generation sequencing case‐control study of 1,263 participants. Front Oncol. 2020;10:666. 32547938 10.3389/fonc.2020.00666 PMC7273971

[35] Sudlow C , Gallacher J , Allen N , et al. UK biobank: an open access resource for identifying the causes of a wide range of complex diseases of middle and old age. PLoS Med. 2015;12(3):e1001779. 25826379 10.1371/journal.pmed.1001779 PMC4380465

[36] Lek M , Karczewski KJ , Minikel EV , et al. Analysis of protein‐coding genetic variation in 60,706 humans. Nature. 2016;536(7616):285‐291. 27535533 10.1038/nature19057 PMC5018207

[37] Naslavsky MS , Yamamoto GL , de Almeida TF , et al. Exomic variants of an elderly cohort of Brazilians in the ABraOM database. Hum Mutat. 2017;38(7):751‐763. 28332257 10.1002/humu.23220

[38] Tunçbílek E , Koc I . Consanguineous marriage in Turkey and its impact on fertility and mortality. Ann Hum Genet. 1994;58(4):321‐329. 7864588 10.1111/j.1469-1809.1994.tb00729.x

[39] Makhnoon S , Levin B , Ensinger M , et al. A multicenter study of clinical impact of variant of uncertain significance reclassification in breast, ovarian and colorectal cancer susceptibility genes. Cancer Med. 2023;12(3):2875‐2884. 36426404 10.1002/cam4.5202 PMC9939195

[40] Makhnoon S , Bednar EM , Krause KJ , Peterson SK , Lopez‐Olivo MA . Clinical management among individuals with variant of uncertain significance in hereditary cancer: a systematic review and meta‐analysis. Clin Genet. 2021;100(2):119‐131. 33843052 10.1111/cge.13966 PMC8672382

[41] Eccles DM , Mitchell G , Monteiro AN , et al. BRCA1 and BRCA2 genetic testing‐pitfalls and recommendations for managing variants of uncertain clinical significance. Ann Oncol. 2015;26(10):2057‐2065. 26153499 10.1093/annonc/mdv278 PMC5006185

[42] Ackerman MJ . Genetic purgatory and the cardiac channelopathies: exposing the variants of uncertain/unknown significance issue. Heart Rhythm. 2015;12(11):2325‐2331. 26144349 10.1016/j.hrthm.2015.07.002

[43] Vos J , Otten W , van Asperen C , Jansen A , Menko F , Tibben A . The counsellees' view of an unclassified variant in BRCA1/2: recall, interpretation, and impact on life. Psychooncology. 2008;17(8):822‐830. 18157792 10.1002/pon.1311

[44] Shaw T , Fok R , Courtney E , Li ST , Chiang J , Ngeow J . Missed diagnosis or misdiagnosis: common pitfalls in genetic testing. Singapore Med J. 2023;64(1):67‐73. 36722519 10.4103/singaporemedj.SMJ-2021-467 PMC9979802

[45] Richter S , Haroun I , Graham TC , Eisen A , Kiss A , Warner E . Variants of unknown significance in BRCA testing: impact on risk perception, worry, prevention and counseling. Ann Oncol. 2013;24(Suppl 8):viii69‐viii74. 24131974 10.1093/annonc/mdt312

[46] Hoffman‐Andrews L . The known unknown: the challenges of genetic variants of uncertain significance in clinical practice. J Law Biosci. 2017;4(3):648‐657. 29868193 10.1093/jlb/lsx038 PMC5965500

[47] O'Neill SC , Rini C , Goldsmith RE , Valdimarsdottir H , Cohen LH , Schwartz MD . Distress among women receiving uninformative BRCA1/2 results: 12‐month outcomes. Psychooncology. 2009;18(10):1088‐1096. 19214961 10.1002/pon.1467 PMC3503506

[48] Mersch J , Brown N , Pirzadeh‐Miller S , et al. Prevalence of variant reclassification following hereditary cancer genetic testing. JAMA. 2018;320(12):1266‐1274. 30264118 10.1001/jama.2018.13152 PMC6233618

[49] Slavin TP , Van Tongeren LR , Behrendt CE , et al. Prospective study of cancer genetic variants: variation in rate of reclassification by ancestry. J Natl Cancer Inst. 2018;110(10):1059‐1066. 29618041 10.1093/jnci/djy027 PMC6249694

[50] Ossa Gomez CA , Achatz MI , Hurtado M , et al. Germline pathogenic variant prevalence among Latin American and US Hispanic individuals undergoing testing for hereditary breast and ovarian cancer: a cross‐sectional study. JCO Glob Oncol. 2022;8:e2200104. 35867948 10.1200/GO.22.00104 PMC9812461

[51] Li D , Shi Y , Li A , et al. Retrospective reinterpretation and reclassification of BRCA1/2 variants from Chinese population. Breast Cancer. 2020;27(6):1158‐1167. 32566972 10.1007/s12282-020-01119-7

[52] Park JS , Nam EJ , Park HS , et al. Identification of a novel BRCA1 pathogenic mutation in Korean patients following reclassification of BRCA1 and BRCA2 variants according to the ACMG standards and guidelines using relevant ethnic controls. Cancer Res Treat. 2017;49(4):1012‐1021. 28111427 10.4143/crt.2016.433 PMC5654176

[53] Plon SE , Rehm HL . The ancestral pace of variant reclassification. J Natl Cancer Inst. 2018;110(10):1133‐1134. 29757403 10.1093/jnci/djy075 PMC6186517

